# Supplementary material for: How social media data are being used to research the experience of mourning: A scoping review
Source: PLoS One. 2022 Jul 22;17(7):e0271034. doi: 10.1371/journal.pone.0271034 (PMC9307163; doi:10.1371/journal.pone.0271034)
Supplement: S2 Appendix — (DOCX) [file pone.0271034.s002.docx]

Multimedia Appendix 2

Search Strategy for CINAHL

| Search platform: CINAHL | |
| --- | --- |
| Limits: none | |
| Search terms: | |
| Mourning | Social Media |
| MH Bereavement+  OR  TI grief  OR  TI bereavement  OR  TI mourning  OR  AB grief  OR  AB bereavement  OR  AB mourning | MH “social media+”  OR  TI “web 2.0“  OR  TI “social media”  OR  TI internet  OR  TI online  OR  TI Facebook  OR  TI myspace  OR  TI Twitter  OR  TI Instagram  OR  TI “social networking”  OR  TI YouTube  OR  TI blog  OR  AB “social media”  OR  AB internet  OR  AB online  OR  AB Facebook  OR  AB myspace  OR  AB Twitter  OR  AB Instagram  OR  AB “social networking”  OR  AB YouTube  OR  AB blog |

CINAHL Search

(MH Bereavement+ OR TI grief OR TI bereavement OR TI mourning OR AB grief OR AB bereavement OR AB mourning) AND (MH “social media+” OR TI “web 2.0“ OR TI “social media” OR TI internet OR TI online OR TI Facebook OR TI myspace OR TI Twitter OR TI Instagram OR TI “social networking” OR TI YouTube OR TI blog OR AB “social media” OR AB internet OR AB online OR AB Facebook OR AB myspace OR AB Twitter OR AB Instagram OR AB “social networking” OR AB YouTube OR AB blog)
