# Supplementary material for: How social media data are being used to research the experience of mourning: A scoping review
Source: PLoS One. 2022 Jul 22;17(7):e0271034. doi: 10.1371/journal.pone.0271034 (PMC9307163; doi:10.1371/journal.pone.0271034)
Supplement: Attachment — Submitted filename: Response to reviewers.docx [file pone.0271034.s004.docx]

| **Comment** | **Reviewer comment** | **Response to reviewer comment** | **Line number, page number** |
| --- | --- | --- | --- |
| **Reviewer #1** |  |  |  |
| Introduction |  |  |  |
| 1 | Social Media and research (page 4): After reading this section, its title seemed a little bit vague and not specific. Also, the authors put diverse content all together in this part, which I thought may lead to some confusion for readers. | Thank you for this observation. On reflection we believe the introduction will read better without subheadings and have now deleted these. We have considered the flow of the introduction, and are satisfied that it now describes the focus, scope and purpose of the review, and provides appropriate definitions for the concepts being explored. | Pages 5-7 |
| 2 | Social Media and research (page 4): In recent years, there have been a variety of social media platforms launched with quite distinct features, such as Instagram best for sharing photos but not lengthy articles. In addition, different platforms may have dissimilar users of demographics and audience targeted, which I suggest bringing some related background/discussions. | Thank you for the suggestion. We have added further content regarding types of platforms and target demographics. | Page 5  (Lines 69-72) |
| 3 | Types of SM data used by researchers (page 6): The part from the lines 86 to 96 “While research using SM data can be valuable in the quest to …” seemed to be sort of not matched to the section’s title. Might consider separating it from the section or strengthening the relevance. | We appreciate the suggestion and have removed subheadings from the introduction, and believe it flows better this way.  Also, the section of the introduction where ethical issues are introduced has been strengthened. | Pages 6-7 |
| 4 | Ethical consideration of social media seems to be one of the topics of interest the authors want to highlight and discuss; suggest having an independent section of it in Introduction. | We have strengthened the section in the introduction regarding ethical considerations re SM. In line with the above we are moving away from subheadings but have added content as per your suggestion. | Page 6 |
| Methodology |  |  |  |
| 5 | (page 8): Because some readers may not be so familiar with the scoping review methodology, it would be greatly helpful if there is still a part ahead briefly describing the whole procedure. | Thank you for the suggestion. We have added further details to the description of scoping reviews. | Page 9 |
| 6 | Eligibility criteria (page 9, line 163): Here I suggest including/listing at least one or two criteria examples. Eligibility criteria (page 9, line 167): The statement “... that had expressed grief on SM” may be quite vague and not concrete to readers; I believe adding some instances that can help make it more concrete. | Thank you for the suggestion. We have included examples of online mourning behaviour. | Page 10  (lines 193 - 195) |
| 7 | Eligibility criteria (page 10, line 184): Why used MEDLINE and CINAHL as the initial search databases? Should provide the considerations. | We were advised by the academic librarian to start with these two databases, and the text has been changed to reflect this. | Page 11 (line 221) |
| 8 | Eligibility criteria (page 11, line 213): Could the authors clarify what “quality control” actually means here? And what were the processes to maintain quality in detail? | Thank you for the suggestion.  Please see clarified statement. | Page 13 (lines 251 -253) |
| Discussion |  |  |  |
| 9 | May have a part highlighting the practical implications for future studies, as well as policy recommendations. If can further improve the connection between paragraphs, this will make readers read the article smoother and easier | Thank you for your time reviewing this manuscript. Your comments and considerations are much appreciated. | Page 29 |
| **Reviewer #2** |  |  |  |
|  | The authors provide a short review on how SM data are being used to study mourning experience. Although the scope of this review is narrow comparing to most review articles, the topic itself is interesting and in frontier. In principle, it has the potential to be accepted, however, the submission has remarkable drawbacks and thus cannot be accepted in the current form. Specific comments and suggestions are as follows. | Thank you for this feedback. |  |
| 1 | This review provides rich materials as well as the concerned issues in related studies, while it lacks description of main findings in the mentioned works. For example, what are the answers to the main question and the four sub-questions. | Thank you for this comment. We have strengthened the conclusion to reflect this. The 4 main questions are explored in results and discussion. Please see edits. | Page 31 |
| 2 | In the review article [J. Gao, Computational Socioeconomics, Physics Reports 2019], the authors introduced how to use SM data to reveal and predict socioeconomic phenomena. In particular, the subsection “Online posts for disease surveillance” described very similar issues to what introduced in this submission. The authors should highlight this review, as well as some closely related but missed works (mainly in the above-mentioned subsection). | Thank you for this suggestion. However, after careful consideration, we found that this suggestion is not directly relevant to the central themes of this review, which are the expression of grief and mourning online. |  |
| 3 | The introduced methodology on SM data is similar to those summarized in the Perspective [T. Zhou, Representative methods of computational socioeconomics, J. Phys. Complexity 2021], in particular, I suggest the authors use “natural data” instead of “naturally occurring data” (how to deal with natural data is one main topic in the above-mentioned Perspective). This review spent a lot on ethical issues, which are highly valuable. In addition to the current discussion, I suggest the authors pay more attention to the private protection problem. Because when natural data is used, the involved individuals are not aware of the fact that they are under investigation. Even if the data is collected from public websites, the private information has to be protected because individuals probably do not want other people knowing the analytic results related to them, or do not want to see the results themselves. For example, a user is willing to share his information to Facebook friends, which does not imply that he agrees to be known as a predicted depression patient or a predicted gay based on his shared data, see for example [M. De Choudhury, et al., AAAI Conf. Web and Social Media 2013] and [M. Kosinski, et al., Private traits and attributes are predictable from digital records of human behavior, PNAS 2013]. | Thank you for the suggestion and the article. The term natural data is now being used instead of naturally occurring data, supported by the suggested paper. | Throughout the review and tables. |
| 4 | Related to Comment [3], the authors should mention an ethical issue that one can use AI or data mining technique to deanonymized data, see for example, the two famous paper by Y.-A. De Montjoye, et al., [Unique in the crowd, Scientific Reports 2013] and [Unique in the shopping mall, Science 2015]. | We appreciate the suggestions and have added these two papers to support content in the introduction. | Page 6 (lines 98 - 99) |
| 5 | For the “researcher generated data” (it is better to use generated data instead), if the researchers intend to interact with subjects (aware or not aware, personally or by some pre-designed SM-robots), researchers have to carefully evaluate in advance whether the materials shown to subjects, the interactions themselves, and the feedback from researchers will result in long-standing negative impacts on subjects’ emotion and mentality. | Thank you for the suggestion. The term generated data is now being used instead of researcher generated data. | Throughout the review and tables. |
| 6 | In a recent work [C. Liu, et al., Emoji use in China: popularity patterns and changes due to COVID-19, Applied Intelligence 2022], emoji usage in SM was analyzed to reveal temporal patterns of sentiments in the outbreak of COVID-19, which is closely related to the COVID-19 issue mentioned in the submission. By the way, the authors should introduce the main findings of Refs. [56] and [99]. | Thank you for the suggestion. The main findings of both studies were added to the review. | Pages 22-23  (Lines 361 - 366) |
